# Peer Review of “Impact of the COVID-19 Pandemic on Routine Childhood Vaccination Coverage in Ecuador From 2019 to 2021: Comparative Analysis”

**DOI:** 10.2196/84849

**Published:** 2025-10-17

**Authors:** Busurat Adenike Mudashiru

**Affiliations:** 1 Ohio University, Athens, OH, United States

**Keywords:** COVID-19 pandemic, vaccination coverage, Ecuador, immunization, routine vaccination, health disparities, vaccine hesitancy

*This is the peer-review report for “Impact of the COVID-19 Pandemic on Routine Childhood Vaccination Coverage in Ecuador From 2019 to 2021: Comparative Analysis.”*

## Round 1 Review

### General Comments

This paper [1] is highly impactful. The authors address a significant gap in current knowledge of the impact of COVID-19 on vaccination coverage.

### Specific Comments

There is a formatting issue; the figures and tables should be neatly placed.

#### Major Comments

1. The tables and figures should be well-labeled and referenced.

2. The limitations of the study are briefly mentioned.

3. The statistical methods should be well-presented.

#### Minor Comments

4. The Methods should be more explanatory.

## References

[1] Sanchez J, Rodriguez AA, Cuello KPM (2025). Impact of the COVID-19 Pandemic on Routine Childhood Vaccination Coverage in Ecuador From 2019 to 2021: Comparative Analysis. JMIRx Med.

